# Ethnobotanical Knowledge, Quality Assessment, and Phytochemical Evaluation of Medicinal Plants Used for Respiratory Ailments at Gamarra Market, Lima, Peru

**DOI:** 10.3390/plants15213421

**Published:** 2026-11-03

**Authors:** Flor Lidia Bustamante-Fustamante, Juan E. Valdiviezo-Campos, María Andrea Serrano Boza, Camila Alessandra Vasquez Ramirez, Jessica Paola Bejarano Suca, Ramiro Fiestas-Jacinto, Michelle Lozada-Urbano, Eulalia V. Tapia, Junior F. Siguas-Peña

**Affiliations:** 1 Research in Natural Products Group, Programa Académico de Farmacia y Bioquímica, Facultad de Ciencias de la Salud, Universidad Privada Norbert Wiener, Lima 15046, Peru; flor.bustamante@uwiener.edu.pe (F.L.B.-F.); juan.valdiviezo@uwiener.edu.pe (J.E.V.-C.); ramiro.fiestas@uwiener.edu.pe (R.F.-J.); 2 Phytoresearchers Students Seedbed, Programa Académico de Farmacia y Bioquímica, Facultad de Ciencias de la Salud, Universidad Privada Norbert Wiener, Lima 15046, Peru; a2024200185@uwiener.edu.pe (M.A.S.B.); 2024200510@uwiener.edu.pe (C.A.V.R.); a2023102871@uwiener.edu.pe (J.P.B.S.); 3 Programa Académico de Nutrición y Dietética, Universidad Privada Norbert Wiener, Lima 15046, Peru; michelle.lozada@uwiener.edu.pe; 4 Grupo de Investigación en Alimentos Funcionales y Desarrollo de Productos (AFYDEP), Laboratorio de Investigación en Alimentos Funcionales y Nutraceúticos (LIAFYN), Universidad Nacional de Barranca, Lima 15169, Peru; evargas@unab.edu.pe

**Keywords:** ethnobotany, medicinal plant markets, traditional medicine, heavy metals, phytochemical screening, antioxidant capacity

## Abstract

Urban medicinal plant markets preserve traditional knowledge, but information on the quality and chemical characteristics of commercial plant materials remains limited. This study combined a descriptive cross-sectional survey of 48 medicinal plant vendors at Gamarra Market, Lima, Peru, with selected physicochemical, elemental, and phytochemical analyses of commercial leaves of *Aristeguietia ballii* (Oliv.) R.M.King & H.Rob., *Borago officinalis* L., *Senecio canescens* (Bonpl.) Cuatrec., and *Eucalyptus globulus* Labill. The survey documented plant names, reported medicinal uses, plant parts, preparation methods, and routes of administration. Laboratory analyses included proximate composition, arsenic (As), lead (Pb), and mercury (Hg) determination, preliminary phytochemical screening, spectrophotometric estimates of total phenolic content (TPC) and total flavonoid content (TFC), and DPPH and ABTS radical-scavenging assays. *A. ballii* had the highest concentrations of As (2.18 mg/kg) and Pb (2.54 mg/kg) among the materials analyzed, while Hg was below the detection limit in all samples. *E. globulus* yielded positive reactions in the greatest number of qualitative screening assays and the highest TPC in its hydroethanolic extract (53.22 ± 1.00 mg GAE/g DS) and TFC in its chloroform-derived extract (21.92 ± 0.41 mg QE/g DS). Hydroethanolic *B. officinalis* had the highest DPPH response (33.39 ± 0.63 mg TE/g DS), whereas aqueous *E. globulus* had the highest ABTS response (63.60 ± 1.19 mg TE/g DS). These findings characterize the interviewed vendors’ reports and the commercial samples analyzed. Convenience sampling and one composite commercial sample per species limit generalizability, and the chemical assays do not establish respiratory efficacy or overall toxicological safety.

## 1. Introduction

Medicinal plants play an important role in various primary health care systems around the world, with particular interest in countries with low- and middle-income economies where access to health services remains limited. According to the World Health Organization, traditional medicine continues to be an important and essential resource in public health, even for the vast majority of the world’s population [1]. For this reason, traditional and urban markets for medicinal plants currently function not only as commercial centers but also as important sociocultural spaces and venues for the transmission of ethnobotanical knowledge, where this knowledge is preserved, exchanged, and passed down from generation to generation [2].

Currently, there is an increase in the production of remedies made from medicinal herbs and spices; this, combined with their rapid adoption by the general public, has led to the commercialization of medicinal plants in local markets. This trend provides the public with an alternative to traditional medicine, thus improving access to therapeutic resources in the community. However, it also raises concerns about the authenticity, quality, safety and traceability of herbal products [3,4]. This is a problem of worldwide scale; many cases of problems such as substitution of species, adulteration, improper handling after harvesting, and contamination with toxic substances in products from medicinal plants are observed, which can lead to a potential loss of therapeutic efficacy and increase the risk to public health [4,5,6].

Ethnobotanical knowledge provides important information on the selection, preparation and therapeutic use of medicinal plants; over the years, this knowledge has contributed to the discovery of various bioactive compounds, many of which are used in the creation of contemporary drugs [7,8]. But urbanization and cultural change threaten the continued existence of these traditions and customs. Therefore, urban markets for medicinal herbs are considered strategic sites to document traditional medicinal practices and, hence, to understand their relationship with the community [9].

The collection of information like documentation on the traditional uses of medicinal plants is of great importance. However, it is essential to evaluate their pharmacognostic quality to ensure their safe and rational use. The pharmacognostic evaluation is useful to assess the physicochemical properties, quality parameters and contamination levels that can alter their efficacy and safety [10,11]. These analyses should be considered essential and important for plants marketed in urban markets, where the differences in harvesting, storage, transportation and marketing conditions can directly influence the quality of the product that arrives in the community.

Phytochemical assays constitute another important aspect of the scientific evaluation of medicinal plants. Secondary metabolites like phenolic compounds, flavonoids, alkaloids, tannins and terpenoids are reported to possess a broad spectrum of biological properties, mainly antioxidant, anti-inflammatory and antimicrobial activities [12]. Total phenolic and flavonoid contents provide general information on important classes of plant secondary metabolites, whereas in vitro assays such as DPPH and ABTS provide complementary information on the radical-scavenging capacity of plant extracts [13]. Although these chemical assays are useful for comparative phytochemical assessment, they do not directly demonstrate biological or therapeutic efficacy.

Previous studies of medicinal plant markets in Asia, Africa and Latin America have emphasised their importance in safeguarding traditional knowledge and linking medicinal plant usage to biodiversity, cultural practices and socio-economic factors [14,15]. In Peru, ethnobotanical studies have documented medicinal plant use, including applications for respiratory disorders [16,17]. An investigation in Trujillo also combined ethnobotanical documentation with measurements of phenolic and flavonoid content [18]. This is particularly relevant in large urban markets, where plant materials may originate from different supply chains and be subject to variable harvesting, processing, transportation and storage conditions [19]. Ethnobotanical documentation alone cannot establish the physicochemical quality, elemental composition, or phytochemical characteristics of the materials sold, whereas laboratory analyses conducted without ethnobotanical context may overlook the traditional uses and preparation practices associated with these products.

To address this gap, the present study aimed to: (i) document vendor-reported medicinal uses, plant species, and preparation practices through a descriptive cross-sectional ethnobotanical survey at Gamarra Market, Lima, Peru; (ii) characterize selected physicochemical quality parameters (proximate composition) and inorganic contaminant concentrations (As, Pb, and Hg) of the four most frequently commercialized species; and (iii) evaluate their preliminary phytochemical composition, total phenolic and flavonoid contents, and DPPH and ABTS radical-scavenging capacities in extracts of different polarities. By integrating these complementary approaches, the study aimed to provide a contextual characterization of medicinal plant materials commercialized at this urban market and to identify priorities for further quality control and safety-oriented investigations, without implying clinical efficacy or representativeness beyond the investigated samples and participants.

## 2. Results

Table 1 summarizes the sociodemographic characteristics of the 48 medicinal plant vendors interviewed at Gamarra Market in Lima, Peru. Women accounted for 56.25% (*n* = 27), while men accounted for 43.75% (*n* = 21). The largest age group was 31–40 years (35.42%), followed by 41–50 years (29.17%), 51–60 years (20.83%) and 61–70 years (14.58%). Most participants reported having completed secondary education (66.67%), followed by primary education (22.92%) and higher education (10.42%). The majority of participants were herbalists (64.58%), followed by apprentices (20.83%), traditional healers (10.42%), and family successors (4.17%).

Figure 1 summarizes vendor-reported medicinal uses, species, growth habits, plant parts, preparation methods, and routes of administration. Respiratory disorders represented the most frequently reported therapeutic applications, with cough accounting for 33.3% of the cited uses, followed by influenza (17.9%), bronchial conditions (16.7%), sore throat (13.1%), phlegm accumulation (9.5%), common cold (4.8%), and asthma (4.7%) (Figure 1A).

Nine medicinal plant species were individually documented among the taxa commercialized at Gamarra Market for respiratory ailments, distributed across seven genera and five botanical families (Table 2). Asteraceae was the most represented family, contributing five species (55.6% of identified taxa): *Aristeguietia ballii* (Oliv.) R.M.King & H.Rob., *Aristeguietia discolor* (DC.) R.M.King & H.Rob., *Senecio canescens* (Bonpl.) Cuatrec., *Senecio coymolachensis* Cabrera, and *Perezia multiflora* (Bonpl.) Less. The remaining families were represented by one species each: Myrtaceae (*Eucalyptus globulus* Labill.), Boraginaceae (*Borago officinalis* L.), Piperaceae (*Piper aduncum* L.), and Polygonaceae (*Muehlenbeckia volcanica* (Benth.) Endl.). Seven of the nine identified species (77.8%) are native to the Andean region, while two (*E. globulus* and *B. officinalis*) are introduced species.

Among the medicinal plants reported by the informants, *Eucalyptus globulus* was the most frequently commercialized species (23.23%), followed by *Aristeguietia ballii* (15.15%), *Senecio canescens* (12.12%) and *Borago officinalis* (11.11%), and other species such as *Piper aduncum* (matico), *Aristeguietia discolor* (pulmonaria), *Senecio coymolachensis* (huamanripa), *Muehlenbeckia volcanica* (mullaca), and *Perezia multiflora* (escorzonera), each representing smaller proportions of the market inventory (Figure 1B).

In terms of growth habit, herbaceous species predominated (50.6%), followed by shrubs (29.4%) and trees (20.0%) (see Figure 1C). In terms of the parts of the plant utilized, the most commonly used organ was leaves (49.4%), followed by the whole plant (21.0%), flowers (18.5%), stems (6.2%) and roots (4.9%) (Figure 1D).

Infusion was by far the most frequently reported method of preparation (78.3%). Other preparation methods such as powder formulations (3.3%), decoctions (5.0%), macerations (5.0%), and miscellaneous preparations (8.4%) were considerably less common (Figure 1E). The most common route of use was oral administration (82.4%), followed by external application (12.3%) and other routes of administration (5.3%) (Figure 1F).

Selected physicochemical measurements differed among the commercial leaf materials analyzed (Table 3). For proximate composition, *Aristeguietia ballii* had the highest moisture content (11.68%) and *Borago officinalis* had the lowest moisture content (6.93%). The ash content ranged from 6.15% in *Senecio canescens* to 15.07% in *A. ballii*, reflecting diversity in the mineral and inorganic fraction of the analyzed commercial products.

Raw protein content ranged from 13.50% to 16.04% with the maximum concentration in *B. officinalis*. Carbohydrate was the major nutritional component in all species, ranging from 39.78% in *A. ballii* to 54.92% in *S. canescens.* The dietary fiber contents ranged from 8.07% to 14.08%, with the highest values found in *B. officinalis* and *Eucalyptus globulus*.

Mercury concentrations were below the detection limit (<0.05 mg/kg) for all samples. Arsenic concentrations were the highest in *A. ballii* (2.18 mg/kg) and *B. officinalis* (0.40 mg/kg), but none of the studied species exceeded the general reference limits used in this study for arsenic (10 mg/kg) and lead (10 mg/kg). However, *Aristeguietia ballii* had the highest levels of both As (2.18 mg/kg) and Pb (2.54 mg/kg) of the four species analyzed. For context, the Pb concentration found in *A. ballii* was also above the maximum level of 2.0 mg/kg set by the Codex Alimentarius for lead in dried culinary herbs. Although this Codex value cannot be used directly as a regulatory limit for medicinal plants, the comparison offers a further reference for the interpretation of the Pb concentration found in this commercial plant material.

Preliminary phytochemical screening revealed significant differences in the metabolite composition of the evaluated medicinal plants (Table 4). *Eucalyptus globulus* exhibited positive reactions in the greatest number of qualitative screening assays, including quinones, coumarins, cardiac glycosides, alkaloids, steroids/triterpenes, carbohydrates and tannins. Strong qualitative reactions (+++) were observed for tannins and coumarins under the assay conditions. *Borago officinalis* was characterized by the presence of phenolic compounds, flavonoids, alkaloids and tannins, with flavonoids showing the strongest reaction. In contrast, *Senecio canescens* demonstrated a predominance of coumarins, alkaloids, proteins, catechins, carbohydrates, and tannins, with particularly intense reactions for proteins, catechins, and carbohydrates.

Among the evaluated species, *Aristeguietia ballii* exhibited the fewest positive reactions in the preliminary phytochemical screening, with a positive reaction observed only in the tannin assay. No detectable reactions were observed for the other metabolite classes under the experimental conditions employed. However, these negative qualitative reactions should not be interpreted as evidence of the complete absence of the corresponding compounds.

Considerable variations were observed in phytochemical composition and radical-scavenging capacity among species and extraction solvents (Table 5). Total phenolic content (TPC) ranged from 0.12 ± 0.00 to 53.22 ± 1.00 mg GAE/g DS. The highest TPC was recorded in the hydroethanolic extract of *Eucalyptus globulus* (53.22 ± 1.00 mg GAE/g DS), followed by the aqueous extract of *Aristeguietia ballii* (37.45 ± 0.70 mg GAE/g DS) and the hydroethanolic extract of *Borago officinalis* (25.75 ± 0.37 mg GAE/g DS). Chloroform extracts generally showed comparatively low TPC values.

Total flavonoid content (TFC) showed a different solvent-dependent pattern. The highest value was observed in the chloroform extract of *E. globulus* (21.92 ± 0.41 mg QE/g DS), followed by the hydroethanolic extract of *Senecio canescens* (8.80 ± 0.13 mg QE/g DS) and the aqueous extract of *E. globulus* (7.85 ± 0.12 mg QE/g DS). However, the relatively high TFC detected in the chloroform extract of *E. globulus* was not consistently observed for the other species.

DPPH radical-scavenging capacity ranged from 1.57 ± 0.03 to 33.39 ± 0.63 mg TE/g DS. The highest value was observed in the hydroethanolic extract of *B. officinalis* (33.39 ± 0.63 mg TE/g DS), followed by the hydroethanolic extract of *A. ballii* (27.80 ± 0.50 mg TE/g DS) and the aqueous extract of *E. globulus* (22.82 ± 0.41 mg TE/g DS). ABTS radical-scavenging capacity was generally higher than that determined by DPPH. The aqueous extract of *E. globulus* exhibited the highest ABTS value (63.60 ± 1.19 mg TE/g DS), followed by its hydroethanolic extract (57.42 ± 1.03 mg TE/g DS), the hydroethanolic extract of *S. canescens* (48.59 ± 0.91 mg TE/g DS), and the aqueous extract of *S. canescens* (44.18 ± 0.80 mg TE/g DS). Overall, aqueous and hydroethanolic extracts generally exhibited greater radical-scavenging capacity than chloroform extracts.

## 3. Discussion

This study combined vendor-reported ethnobotanical information with selected analyses of commercial medicinal plant leaves sourced from Gamarra Market. These two components address complementary questions: the survey describes reported uses and practices, while the laboratory measurements characterize the analyzed materials. Neither component establishes therapeutic efficacy.

Consistent with studies highlighting their role in preserving and transmitting traditional medicinal knowledge, women represented 56.3% of the surveyed vendors [16,21,22,23]. However, gender-related patterns can vary across sociocultural settings [24], so the predominance of women observed in this market should not be generalized beyond the surveyed participants. Most vendors were aged 31–60 years (85.4%), whereas only 4.2% identified themselves as family successors. While these findings do not demonstrate intergenerational knowledge loss, they emphasize the importance of documenting traditional practices in urban environments, where knowledge transmission may be influenced by migration, apprenticeship and socioeconomic change [2,25,26].

Respiratory conditions accounted for over 90% of the reported therapeutic uses, particularly for treating coughs, influenza, bronchial conditions, sore throats, phlegm, the common cold and asthma. Respiratory uses of medicinal plants have also been documented in Peru [16,17] and Nigeria [27]. The reported uses of the selected species also broadly correspond to previous literature. For example, eucalyptus species are widely used for respiratory complaints, and preparations containing 1,8-cineole have been investigated for their respiratory effects [28,29,30,31]. A systematic review and meta-analysis of six randomized controlled trials involving 1857 participants reported improvements in cough outcomes with eucalyptus-based products compared with placebo [32]. *Borago officinalis* is traditionally used for respiratory conditions, and its raw leaf extracts have demonstrated relaxation of isolated tracheal smooth muscle [33]; and *Senecio canescens* has been reported in Peruvian and Bolivian traditional medicine for related complaints [34]. Nevertheless, evidence concerning specific preparations or extracts should not be extrapolated to the commercial plant materials evaluated in this study, for which respiratory pharmacological activity was not investigated.

Herbaceous plants predominated among the reported growth forms (51%), and leaves were the most frequently used plant part (49%), consistent with ethnobotanical studies emphasizing their accessibility and availability [15,27]. Plant secondary metabolites have documented roles in stress and defense responses [35] and provide opportunities for drug discovery [36]. Infusion (79%) and oral administration (83%) were the predominant preparation and administration practices, respectively. The predominance of infusions provides a relevant ethnobotanical context for evaluating aqueous extracts, although the laboratory extraction conditions do not necessarily reproduce traditional household preparations.

Published conservation and distribution records provide additional context for the taxa documented in this survey. Plants of the World Online records *Piper aduncum* L. as Least Concern (LC), based on an IUCN Red List assessment [37], whereas *Senecio coymolachensis* Cabrera is reported as endemic to Peru [38]. These classifications describe species-level conservation and distribution information and do not establish the geographic origin or harvesting conditions of the commercial materials examined.

The physicochemical assessment revealed differences among the evaluated commercial materials. Moisture contents ranged from 6.50% to 11.68%, with *A. ballii* exhibiting the highest moisture (11.68%) and total ash (15.07%) values. Moisture control is relevant to the storage stability of dried herbal materials [39], whereas total ash reflects both endogenous mineral constituents and potentially extraneous inorganic material introduced during harvesting, processing, or handling [39]. Carbohydrates constituted the largest reported proximate fraction, followed by protein and dietary fiber [40]. These parameters provide compositional information, particularly for plants with food–medicine applications such as *B. officinalis* [41], but do not establish therapeutic value.

The proximate composition results provide descriptive information on the commercial leaf materials analyzed. These measurements complement the selected quality assessments but do not establish nutritional benefits or therapeutic efficacy. Because only one composite commercial sample per species was analyzed, the observed differences should not be generalized to other commercial batches or to the species as a whole.

The As, Pb, and Hg measurements provide an important safety-related component of the present study because medicinal plants may accumulate inorganic contaminants through environmental exposure or post-harvest handling [39,42]. None of the evaluated materials exceeded the general reference concentrations of 10 mg/kg adopted in this study for As and Pb [20], and Hg remained below the analytical detection limit in all samples. Elemental contamination has also been investigated in herbal products [5] and Peruvian food and medicinal products [43]. Nevertheless, *A. ballii* exhibited the highest As (2.18 mg/kg) and Pb (2.54 mg/kg) concentrations. Environmental contamination can influence elemental concentrations in plants [44], although the sources of contamination were not determined in this study. The comparatively higher ash, As, and Pb values observed in *A. ballii* warrant further investigation of elemental contamination and quality variability. Importantly, the results obtained from a single composite commercial sample per species cannot establish the prevalence of contamination or a direct toxicological risk.

Preliminary phytochemical screening revealed differences in qualitative assay responses among the investigated species. *E. globulus* exhibited positive reactions in the greatest number of qualitative screening assays, including strong reactions for tannins and coumarins. Published studies have characterized bioactive constituents of *E. globulus* leaf extracts [29], but they do not confirm the identity of compounds responsible for the qualitative reactions observed here. Published phytochemical work on *B. officinalis* provides species-specific context [45], although differences in extracts and methods limit direct comparison. Nevertheless, these screening assays provide preliminary indications of metabolite classes rather than compound-specific identification or quantitative characterization. This limitation is particularly relevant to pyrrolizidine alkaloids (PAs), which have been reported in *B. officinalis* and commercial material marketed as asmachilca [46,47,48]. Reports concerning commercial asmachilca do not establish the presence of PAs in the authenticated *A. ballii* material analyzed here. Negative or weak reactions with general alkaloid reagents, including Dragendorff’s, Wagner’s, and Mayer’s reagents, cannot exclude the presence of specific alkaloid subclasses. Because PAs were not specifically measured in the present study, their presence or concentration in the analyzed materials remains unknown. Targeted chromatographic techniques, such as LC–MS/MS, would be required to investigate these compounds in future safety assessments.

The qualitative phytochemical screening provides preliminary indications of metabolite classes rather than compound-specific identification or a measure of chemical diversity. Reaction intensities should not be interpreted as quantitative differences in metabolite concentrations, and negative reactions do not establish compound absence. Similarly, TPC and TFC are operational spectrophotometric estimates expressed as gallic acid and quercetin equivalents, respectively, rather than absolute concentrations of identified constituents. DPPH and ABTS assays measure chemical radical-scavenging capacity under the specified experimental conditions. Comparisons among extracts obtained using solvents of different polarities require caution because extraction selectivity, constituent solubility, and assay-specific reactivity may influence the responses. Although the chloroform extracts were evaporated and reconstituted in absolute ethanol before analysis, this solvent exchange does not eliminate compositional differences among the extracted fractions. Accordingly, these findings support descriptive comparisons of the analyzed preparations and should not be interpreted as evidence of biological antioxidant effects or respiratory therapeutic efficacy.

The phytochemical measurements varied among species and extraction solvents. Hydroethanolic extracts generally exhibited higher total phenolic content (TPC), consistent with the ability of polar and intermediate-polarity solvent systems to recover phenolic constituents [49,50,51]. The highest TPC was recorded in the hydroethanolic extract of *E. globulus* (53.22 ± 1.00 mg GAE/g DS), in agreement with previous evidence concerning solvent-dependent phenolic recovery in this species [28]. In contrast, the highest total flavonoid content (TFC) was observed in the chloroform extract of *E. globulus* (21.92 ± 0.41 mg QE/g DS), a pattern not consistently observed in the other species. Therefore, this result should not be interpreted as evidence of generally superior flavonoid extraction by chloroform. Published studies of *B. officinalis* extracts provide further context for its phenolic constituents and antioxidant responses [45,52], although extraction conditions differ and limit direct numerical comparison. These comparisons remain descriptive because the experimental replicates originated from separate extractions of the same composite commercial material rather than independent commercial batches.

Aqueous and hydroethanolic extracts generally showed higher DPPH and ABTS radical-scavenging capacities than chloroform extracts [52,53]. ABTS values were generally higher than DPPH values, potentially reflecting differences in radical chemistry, reaction conditions, solvent compatibility, and constituent reactivity [54]. Notably, TPC, TFC, and radical-scavenging responses did not follow identical patterns: hydroethanolic *E. globulus* exhibited the highest TPC, hydroethanolic *B. officinalis* showed the highest DPPH value (33.39 ± 0.63 mg TE/g DS), and aqueous *E. globulus* exhibited the highest ABTS value (63.60 ± 1.19 mg TE/g DS). These differences suggest that the assay responses cannot be attributed solely to total phenolic or flavonoid content and may also reflect constituent composition and assay-specific reaction mechanisms. Accordingly, TPC, TFC, DPPH, and ABTS should be interpreted as complementary analytical measurements. The chemical radical-scavenging responses observed here do not demonstrate antioxidant effects in biological systems or antitussive, expectorant, bronchodilatory, anti-inflammatory, or clinical efficacy. Accordingly, the findings should be interpreted as complementary phytochemical evidence rather than experimental validation of the respiratory uses reported by the interviewed vendors.

Several limitations should be acknowledged. First, the analytical component was based on one homogenized composite commercial sample per species. Three separate extractions were performed for each species–solvent combination using independently weighed portions of that sample. These replicates characterize extraction-level variability but do not capture variability among independent commercial batches. Consequently, the findings describe the materials analyzed and cannot establish species-level characteristics or represent the range of products commercialized in the market. Further studies incorporating independently sourced commercial batches are needed to assess the reproducibility and generalizability of these findings. Second, the ethnobotanical component involved 48 vendors recruited through non-probability convenience sampling at a single market. Therefore, the reported frequencies describe the knowledge and practices of the interviewed vendors and cannot be generalized to all medicinal plant vendors in Lima or to consumers. The self-reported information was not independently verified through observation of preparation or consumption practices, and quantitative ethnobotanical indices, including use value (UV), informant consensus factor (ICF), and fidelity level (FL), were not calculated because the study focused on descriptive documentation of vendor-reported knowledge and practices. Third, the analytical scope did not include targeted PA identification or quantification, detailed anatomical or molecular authentication, or the full range of classical pharmacognostic quality parameters, including foreign matter, specific ash values, and extractive values. Although botanical identity was established through specialist taxonomic authentication and voucher deposition, further complementary authentication and chemical analyses would strengthen the assessment. Future studies should incorporate independently sourced commercial batches, broader ethnobotanical sampling, targeted contaminant analyses, and appropriate pharmacological investigations to evaluate the consistency and relevance of these findings. Moreover, with 48 informants recruited at a single market through convenience sampling, the study has limited statistical power for detecting associations between vendor characteristics and ethnobotanical knowledge patterns; larger and more diverse samples across multiple markets would be necessary to draw generalisable conclusions. In addition, only four species were selected for analytical characterisation, representing a fraction of the total taxa documented in the survey; thus, the phytochemical and quality findings cannot be extrapolated to other species commercialised at the market.

## 4. Materials and Methods

### 4.1. Reagents and Chemicals

Ethanol, chloroform and distilled water were used as extraction solvents. 2,2-diphenyl-1-picrylhydrazyl (DPPH), 2,2-azinobis-(3-ethylbenzothiazoline-6-sulfonic acid) (ABTS), potassium persulfate (K_2_S_2_O_8_), Trolox (6-hydroxy-2,5,7,8-tetramethylchroman-2-carboxylic acid), gallic acid, and quercetin were used as reference standards.

### 4.2. Ethnobotanical Study

A descriptive cross-sectional ethnobotanical study was conducted among medicinal plant vendors at Gamarra Market, La Victoria District, Lima, Peru, between September and October 2025. A total of 48 herbal vendors were recruited using convenience sampling based on their availability and willingness to participate during the survey period. Eligible participants were vendors who commercialized medicinal plants at the study site, were aged 18 years or older, had knowledge of the medicinal plants they sold, and voluntarily agreed to participate in the study. Vendors who did not provide written informed consent or were unable to complete the interview were excluded. Because a non-probability convenience sampling strategy was employed, the surveyed vendors were not intended to constitute a statistically representative sample of all medicinal plant vendors in Lima. Accordingly, the ethnobotanical findings were interpreted descriptively and restricted to the participants and market setting investigated.

Ethnobotanical knowledge was collected through face-to-face semi-structured interviews using a standardized questionnaire. Before each interview, participants were informed of the purpose and procedures of the study and written informed consent was obtained. Interviews were conducted in Spanish and lasted about 10–20 min. The questionnaire provided data on the vernacular names of medicinal plants, reported medicinal uses, plant parts used, condition of use (fresh or dried), methods of preparation and administration, dosage, combinations with other plants and sources of ethnobotanical knowledge. The ethnobotanical survey was performed according to the methodological framework proposed by Valdiviezo-Campos et al. [18]. Ethnobotanical indices were not calculated as the study was mainly aimed at descriptive documentation of knowledge and practices reported by medicinal plant vendors.

The study protocol was approved by the Institutional Ethics Committee of Universidad Norbert Wiener (approval No. A0034-2025; approved on 22 May 2025) and all procedures involving human participants were performed in accordance with the principles of the Declaration of Helsinki.

### 4.3. Plant Material and Sample Preparation

Fresh commercial materials of four frequently commercialized medicinal plant species were purchased from herbal vendors at Gamarra Market, La Victoria District, Lima, Peru (12.06234° S, 77.01227° W) (Figure 2), in October 2025 for selected physicochemical, elemental, and phytochemical analyses. These coordinates identify the point of commercial acquisition rather than the original cultivation or wild-collection localities of the plants.

Botanical identification was supported by reference specimens deposited at *Herbarium* San Marcos (USM), Museo de Historia Natural, Universidad Nacional Mayor de San Marcos, Lima, Peru. The species and corresponding accession numbers were *Aristeguietia ballii* (Oliv.) R.M.King & H.Rob. (“asmachilca,” USM 358684), *Senecio canescens* (Bonpl.) Cuatrec. (“oreja de conejo,” USM 358677), *Borago officinalis* L. (“borraja,” USM 358673), and *Eucalyptus globulus* Labill. (“eucalipto,” USM 358674). Scientific names were checked against Plants of the World Online (POWO; https://powo.science.kew.org; accessed on 23 October 2026). Table 2 provides complementary taxonomic and vendor-reported ethnobotanical information.

Leaves were used for all laboratory analyses of the four species (Figure 3). The plant parts listed in Table 2 refer to vendor-reported uses, which include aerial parts of *S. canescens* and leaves and flowers of *B. officinalis*. These ethnobotanical records therefore describe traditional practices rather than the composition of the laboratory samples.

The fresh materials were transported to the Pharmaceutical Research Laboratory of Universidad Norbert Wiener, where the leaves were washed with 0.05% sodium hypochlorite solution, thoroughly rinsed with distilled water, and air-dried at room temperature. The leaves were subsequently oven-dried at 40 °C to constant weight, ground into powder, and stored in airtight containers protected from light and moisture until analysis.

### 4.4. Preparation of Plant Extracts

Three independent extractions were performed for each plant species using chloroform, 70% ethanol, and distilled water. For each extraction, 1.0 g of dried and powdered plant material was mixed with 10 mL of the corresponding solvent (1:10, *w*/*v*). Each plant–solvent mixture was heated under reflux for 10 min using a temperature-controlled hot plate equipped with a reflux condenser. The heating conditions were adjusted according to the solvent employed, while the reflux system was used to condense solvent vapors and minimize solvent losses during extraction.

For each species–solvent combination, three separately weighed portions of the same homogenized composite commercial leaf sample were subjected to separate extraction procedures using the same solvent (*n* = 3 extraction replicates). Each resulting extract was analyzed separately. These technical replicates characterize variability associated with extraction and subsequent analysis; they do not represent independent biological specimens or commercial batches.

Before the TPC, TFC, DPPH, and ABTS assays, the chloroform extracts were evaporated, and the residues were redissolved in absolute ethanol to restore the original volume of 10 mL per gram of dry plant material, corresponding to a dry-plant-material-equivalent concentration of 100 mg/mL. The reconstituted solutions were visually homogeneous and remained homogeneous after addition of the assay reagents.

For each assay, solvent-matched blanks contained the corresponding volume of absolute ethanol in place of the extract. Extract-specific blanks were also prepared by omitting the signal-generating reagent and maintaining the final assay volume to account for the intrinsic absorbance of the extracts. Blank readings were used to correct the absorbance measurements according to the calculation procedure for each assay.

### 4.5. Selected Physicochemical and Elemental Analyses

The assessment comprised proximate composition and determination of As, Pb, and Hg. Proximate measurements included moisture, ash, crude protein, crude fat, total dietary fiber, and available carbohydrates. These measurements characterized selected physicochemical attributes and elemental contaminants in the commercial leaf materials. Detailed macroscopic and microscopic characterization, foreign matter, acid-insoluble ash, water-soluble ash, and extractive values were outside the analytical scope.

#### 4.5.1. Proximate Composition Analysis

Proximate composition analysis was performed following the methodology described by Culetu et al. [55], with all determinations carried out in triplicate according to the Official Methods of Analysis of AOAC International [56]. All weighings were performed on an analytical balance (Adam Equipment, model LAB 214i, Milton Keynes, UK; readability: 0.1 mg, capacity: 210 g, with internal and external calibration). Samples were cooled between drying and weighing steps in a desiccator charged with silica gel.

Moisture content was determined by the gravimetric loss-on-drying method (AOAC 934.01) [56]. Approximately 5 g of ground dried leaf sample was placed in a pre-tared weighing container and dried to constant weight in a forced-convection oven (SLW 400 SMART, POL-EKO Aparatura, Wodzisław Śląski, Poland). Moisture content was calculated from the mass loss and expressed as a percentage of the initial sample mass.

Ash content was determined by the direct incineration method (AOAC 923.03) [56]. Approximately 5 g of sample was weighed into porcelain crucibles previously ignited, cooled in a desiccator, and tared. Crucibles were placed in a muffle furnace (THERMCONCEPT, model KL 15, Bremen, Germany) at 550 °C until a light gray ash of constant weight was obtained. After cooling in a desiccator, crucibles were promptly reweighed and ash content was expressed as a percentage of the initial sample weight.

The raw protein content was determined by the Kjeldahl method (AOAC 979.09) [56] using a two-unit system from VELP Scientifica (Usmate Velate, Italy). The sample (about 5 g) was digested in a block digester (VELP Scientifica, model DKL) with concentrated sulfuric acid and a catalyst mixture to convert organic nitrogen into ammonium sulfate. The digest was then moved to an automated steam distillation unit (VELP Scientifica, model UDK 139) where sodium hydroxide was added to release ammonia, which was captured by back titration with a standardized acid solution. Total nitrogen was converted to raw protein by using a nitrogen-to-protein conversion factor of 6.25.

Raw fat content was determined by the acid hydrolysis method (AOAC 922.06) [56] using a semi-automatic solvent extraction system operating on the Randall principle (VELP Scientifica, model SER 148). Approximately 5 g of sample was subjected to acid hydrolysis with hydrochloric acid to release lipids bound to proteins and carbohydrates. The hydrolyzed sample was then extracted with petroleum ether in three sequential phases (immersion in boiling solvent, rinsing with cold refluxing solvent, and solvent recovery). The solvent was evaporated and the lipid residue was dried to constant weight and quantified gravimetrically. Results were expressed as a percentage of the initial sample weight.

Total dietary fiber (TDF) was determined by the enzymatic-gravimetric method (AOAC 985.29) [56] using a commercial assay kit (Total Dietary Fiber Kit, ref. 1.12979, Merck KGaA, Darmstadt, Germany), following the manufacturer’s instructions. Duplicate portions of approximately 5 g of dried, defatted sample were gelatinized with heat-stable α-amylase, then sequentially digested with protease and amyloglucosidase to remove residual starch and protein. Four volumes of 95% ethyl alcohol were added to precipitate soluble dietary fiber. The total residue was collected by filtration and washed successively with 78% ethyl alcohol, 95% ethyl alcohol, and acetone.

Proximate composition results were expressed as percentages of the sample mass as analyzed, including residual moisture. Available carbohydrate content was calculated by difference for each replicate as follows: available carbohydrates (%) = 100 − [moisture (%) + ash (%) + crude protein (%) + crude fat (%) + total dietary fiber (%)]. The resulting values were summarized as mean ± standard deviation.

#### 4.5.2. Elemental Contaminant Analysis

The heavy metal content of medicinal plant samples was analyzed to evaluate their safety for human consumption. The concentrations of arsenic (As), lead (Pb) and mercury (Hg) were analyzed by the accredited analytical laboratory, the Servicio de Analysis Tecnológico (SAT). Prior to analysis, the dried and powdered plant samples were subjected to acid digestion in accordance with standardized procedures suitable for plant matrices. Heavy metal determination was performed using inductively coupled plasma mass spectrometry (ICP-MS) under validated operating conditions. The analytical protocol followed the acid digestion approach described by Karadede and Ünlü [57], adapted in accordance with a validated ICP-MS method specifically developed for determining heavy metals in medicinal plant raw materials [58]. Quality control measures included the use of reagent blanks, calibration with certified reference standards, and duplicate analyses to ensure accuracy and precision. Heavy metal concentrations were expressed as mg/kg of dry matter and compared with general reference concentrations reported in the literature for contextual interpretation [20].

### 4.6. Phytochemical Evaluation

#### 4.6.1. Preliminary Qualitative Screening

Preliminary qualitative screening was conducted to detect major classes of secondary metabolites. Plant material was successively extracted with chloroform, 70% ethanol, and water. Standard qualitative tests were performed to identify oils and fats, reducing sugars, resins, saponins, sterols, triterpenes, phenolics, tannins, flavonoids, anthocyanins, free amino acids or amines, alkaloids, coumarins, quinones, mucilage, and catechins, following established protocols [59].

#### 4.6.2. Determination of Total Phenolic and Flavonoid Contents

Total phenolic content (TPC) was determined using the Folin–Ciocalteu colorimetric method, following the procedure described by Valdiviezo-Campos et al. [60], with minor modifications. Briefly, 0.1 mL of each plant extract was transferred to a 10 mL volumetric flask and mixed with 2.0 mL of Folin–Ciocalteu reagent previously diluted 1:10 (*v*/*v*) with distilled water. Subsequently, 4.4 mL of 7.5% (*w*/*v*) sodium bicarbonate solution was added, and the final volume was adjusted to 10 mL with distilled water. A reagent blank was prepared under the same conditions without the plant extract. The reaction mixtures were incubated for 60 min in the dark at room temperature, after which absorbance was measured at 765 nm using a UV–visible spectrophotometer (Peak Instruments C7000V, Houston, TX, USA). Quantification was performed using an external calibration curve prepared with gallic acid over the concentration range of 1.26–10.08 μg/mL. Each analytical determination was performed in triplicate, and the results were expressed as milligrams of gallic acid equivalents per gram of dry plant material (mg GAE/g DS).

Total flavonoid content (TFC) was determined using the aluminum chloride colorimetric method described by Valdiviezo-Campos et al. [60], with minor modifications. Briefly, 0.2 mL of each plant extract was transferred to a 10 mL volumetric flask and mixed with 1.5 mL of distilled water. Subsequently, 0.4 mL of 10% (*w*/*v*) aluminum chloride solution and 0.4 mL of 1 M sodium acetate solution were added, and the final volume was adjusted to 10 mL with distilled water. The reaction mixtures were incubated for 30 min in the dark at room temperature, after which absorbance was measured at 430 nm using a UV–visible spectrophotometer (Peak Instruments C7000V). Quantification was performed using an external calibration curve prepared with quercetin over the concentration range of 1–16 μg/mL. Each analytical determination was performed in triplicate, and the results were expressed as milligrams of quercetin equivalents per gram of dry plant material (mg QE/g DS).

#### 4.6.3. In Vitro Radical-Scavenging Capacity

DPPH radical-scavenging capacity was assessed using a spectrophotometric assay based on the method described by Valdiviezo-Campos et al. [61], originally developed by Brand-Williams et al. [62], with minor modifications. A 0.1 mM DPPH solution was prepared in 96% ethanol. As described above, each extract was prepared from 1.0 g of dry plant material using 10 mL of the corresponding extraction solvent, and the final extract volume was adjusted to 10 mL, corresponding to a dry-material-equivalent concentration of 100 mg/mL. For the assay, a 10 µL aliquot of each extract, corresponding to 1.0 mg of dry plant material, was transferred to a 10 mL volumetric flask, and the volume was completed with the DPPH solution. Thus, the final dry-material-equivalent concentration in the reaction mixture was 0.10 mg/mL. The mixture was gently shaken and incubated in the dark for 30 min, after which absorbance was measured at 517 nm using a Peak Instruments C7000V UV–Vis spectrophotometer. Trolox (3–30 µM/mL) was used as the reference standard. Radical-scavenging capacity was calculated from the Trolox calibration curve, taking into account the amount of dry plant material represented in the reaction mixture, and expressed as milligrams of Trolox equivalents per gram of dry plant material (mg TE/g DS). The assay was independently performed on each of the three separately prepared extracts (*n* = 3), and the results are expressed as mean ± SD.

ABTS radical-scavenging capacity was evaluated using the spectrophotometric method described by Valdiviezo-Campos et al. [61], based on the original procedure of Re et al. [63]. The ABTS radical solution was prepared by mixing equal volumes of 7 mM ABTS and 2.45 mM potassium persulfate (K_2_S_2_O_8_), followed by incubation in the dark for 16 h. Before use, the resulting solution was diluted with 50% ethanol to obtain an absorbance of 0.90 ± 0.02 at 734 nm. Each plant extract had a dry-material-equivalent concentration of 100 mg/mL. For the assay, a 10 µL aliquot of extract, corresponding to 1.0 mg of dry plant material, was transferred to a 10 mL volumetric flask, and the volume was completed with the ABTS radical solution, resulting in a final dry-material-equivalent concentration of 0.10 mg/mL. The mixture was incubated in the dark for 30 min, and absorbance was subsequently measured at 734 nm. Trolox (3–20 µM/mL) was used as the reference standard. Radical-scavenging capacity was calculated from the Trolox calibration curve, taking into account the amount of dry plant material represented in the reaction mixture, and expressed as milligrams of Trolox equivalents per gram of dry plant material (mg TE/g DS). The assay was independently performed on each of the three separately prepared extracts (*n* = 3), and the results are expressed as mean ± SD.

### 4.7. Statistical Analysis

Ethnobotanical data were summarized using frequencies and percentages. The denominator used for each variable or response category was specified in the corresponding table or figure.

Data are presented as mean ± standard deviation (SD) of three independently prepared extracts (*n* = 3) for each species–solvent combination. Each extraction was performed using a separately weighed portion of the same homogenized composite commercial plant sample. Accordingly, the replicates were considered extraction-level experimental replicates rather than independent biological or commercial samples. Differences among extraction solvents and plant species were evaluated descriptively, and no inferential statistical comparisons were used to generalize the findings beyond the analyzed composite samples. Statistical analyses were performed using GraphPad Prism version 8.0 (GraphPad Software, San Diego, CA, USA).

## 5. Conclusions

This study documented vendor-reported knowledge of medicinal plants used for respiratory ailments at Gamarra Market, Lima, Peru, and provided selected physicochemical, elemental, and preliminary phytochemical data for commercial leaf materials of four frequently reported species. The analyzed materials exhibited differences in proximate composition, elemental concentrations, qualitative screening responses, and solvent-dependent TPC, TFC, and DPPH and ABTS radical-scavenging capacities. These findings constitute a characterization of selected attributes rather than a comprehensive pharmacognostic evaluation.

Among the materials analyzed, *Aristeguietia ballii* exhibited the highest concentrations of As (2.18 mg/kg) and Pb (2.54 mg/kg). However, these measurements only characterize selected elemental contaminants and do not establish overall toxicological safety. Previous reports of pyrrolizidine alkaloids in commercial asmachilca material and *Borago officinalis* raise additional safety concerns. However, these compounds were not analysed specifically, so their presence and concentrations in the evaluated samples remain unknown. Negative qualitative alkaloid reactions cannot confirm their absence.

The ethnobotanical findings are restricted to the 48 interviewed vendors, while the analytical findings characterize one composite commercial sample per species. Extraction replicates do not capture between-batch variability. TPC and TFC are spectrophotometric estimates expressed as reference-standard equivalents, and DPPH and ABTS responses do not demonstrate biological antioxidant effects or therapeutic efficacy against respiratory diseases. Further studies should incorporate independently sourced commercial batches, targeted constituent and contaminant analyses, and appropriate pharmacological investigations.

## Figures and Tables

**Figure 1:**
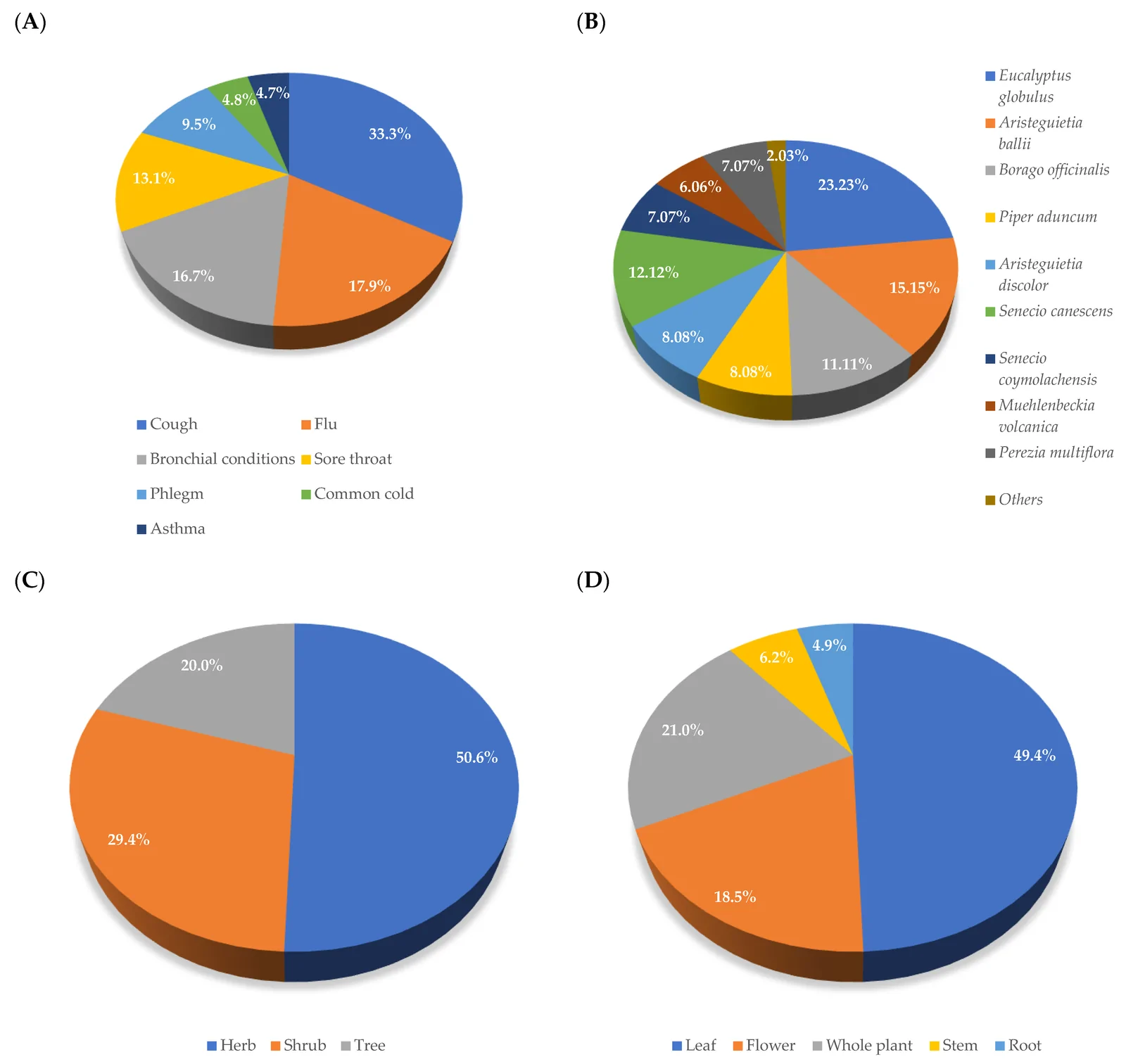

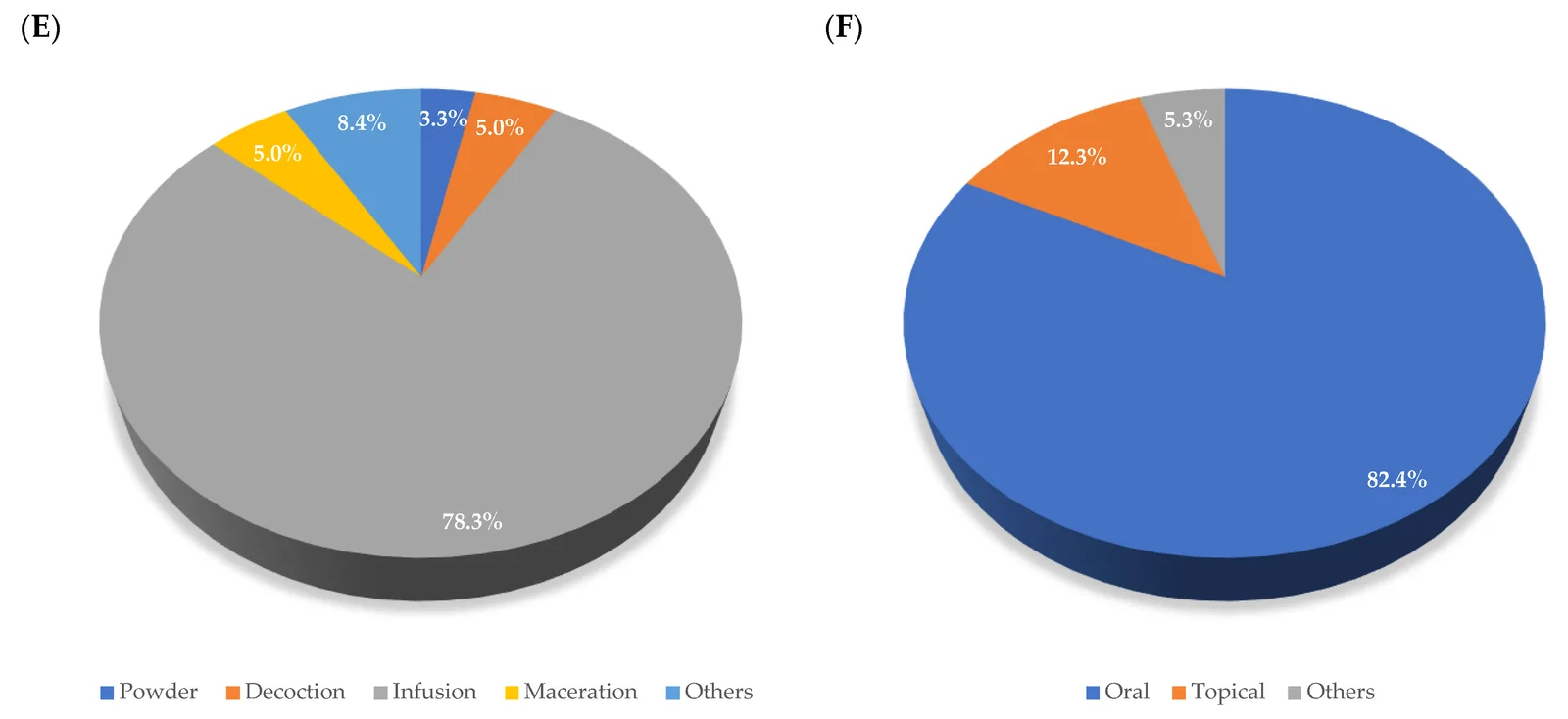
Ethnobotanical documentation of medicinal plants used for respiratory ailments and commercialized at Gamarra Market, Lima, Peru: medicinal uses (**A**), most frequently traded species (**B**), growth habit (**C**), plant parts used (**D**), preparation methods (**E**), and routes of administration (**F**).

**Figure 2:**
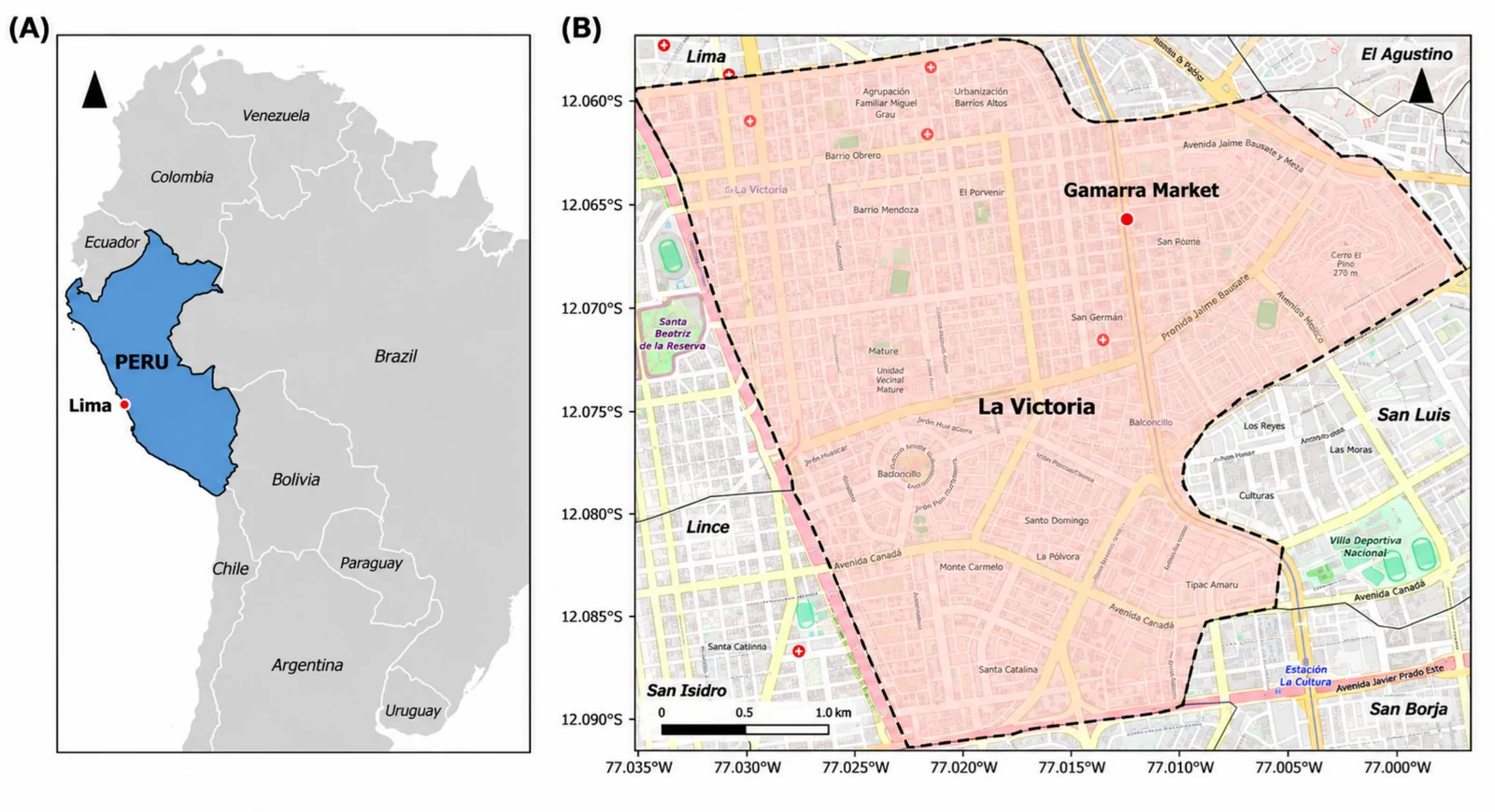
Location of the study area: (**A**) geographic location of Peru within South America, highlighting Lima; (**B**) study area in La Victoria district, Lima, Peru, showing the location of Gamarra Market.

**Figure 3:**
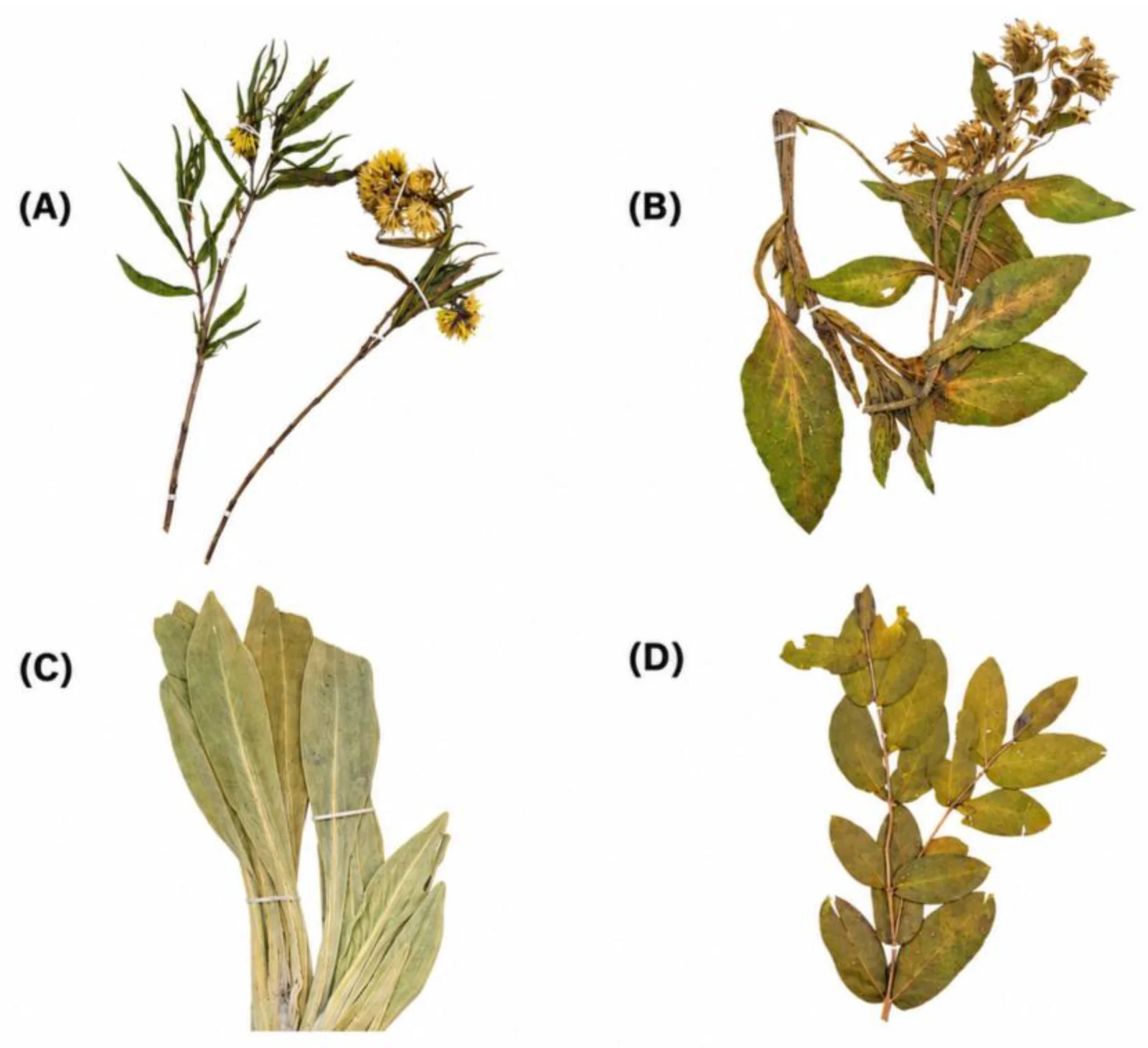
Selected medicinal plant species most frequently commercialized at Gamarra Market: (**A**) *Aristeguietia ballii* (asmachilca), (**B**) *Borago officinalis* (borage), (**C**) *Senecio canescens* (rabbit’s ear), and (**D**) *Eucalyptus globulus* (eucalyptus).

**Table 1:** Sociodemographic characteristics of the informants who market medicinal plants.

Variable	Category	*n* (%)
Gender	Male	21 (43.75)
	Female	27 (56.25)
Age (years)	31–40	17 (35.42)
	41–50	14 (29.17)
	51–60	10 (20.83)
	61–70	7 (14.58)
Educational level	Primary education	11 (22.92)
	Secondary education	32 (66.67)
	Higher education	5 (10.4)
Self-reported role	Traditional healer	5 (10.42)
	Herbalist	31 (64.58)
	Apprentice	10 (20.83)
	Family successor	2 (4.17)

**Table 2:** Floristic inventory of medicinal plant species commercialized at Gamarra Market, La Victoria District, Lima, Peru.

No.	Family	Scientific Name	Local Name	Medicinal Use	Part Used
1	Asteraceae	*Aristeguietia ballii* (Oliv.) R.M.King & H.Rob.	Asmachilca	Anti-inflammatory; analgesic	Leaves
2	Asteraceae	*Aristeguietia discolor* (DC.) R.M.King & H.Rob.	Pulmonaria	Digestive; anti-inflammatory	Leaves
3	Asteraceae	*Perezia multiflora* (Bonpl.) Less.	Escorzonera	Digestive; hepatoprotective	Root
4	Asteraceae	*Senecio canescens* (Bonpl.) Cuatrec.	Oreja de conejo	Respiratory; anti-inflammatory	Aerial parts
5	Asteraceae	*Senecio coymolachensis* Cabrera	Huamanripa	Anti-inflammatory	Aerial parts
6	Boraginaceae	*Borago officinalis* L.	Borraja	Antipyretic; diuretic	Leaves, flowers
7	Myrtaceae	*Eucalyptus globulus* Labill.	Eucalipto	Respiratory; antiseptic	Leaves
8	Piperaceae	*Piper aduncum* L.	Matico	Wound healing; anti-inflammatory	Leaves
9	Polygonaceae	*Muehlenbeckia volcanica* (Benth.) Endl.	Mullaca	Anti-inflammatory; analgesic	Aerial parts

**Table 3:** Selected pharmacognostic quality and safety parameters of the most frequently commercialized medicinal plants at Gamarra Market, Lima, Peru.

Parameter	*Aristeguietia ballii* (Asmachilca)	*Borago officinalis* (Borraja)	*Senecio canescens* (Oreja de Conejo)	*Eucalyptus globulus* (Eucalipto)
Proximate composition				
Moisture	11.68 ± 0.04	6.93 ± 0.05	10.04 ± 3.46	6.50 ± 0.02
Ash	15.07 ± 0.03	10.78 ± 0.11	6.15 ± 1.07	7.22 ± 0.00
Protein raw	14.43 ± 0.14	16.04 ± 2.17	13.50 ± 0.07	14.53 ± 0.08
Fats raw	10.00 ± 0.30	6.80 ± 0.21	7.00 ± 0.12	6.00 ± 0.19
Dietary fiber	8.07 ± 0.30	14.08 ± 0.21	8.39 ± 0.12	13.33 ± 0.19
Carbohydrates	40.75 ± 3.46	45.37 ± 2.19	54.92 ± 3.63	52.42 ± 0.20
Heavy metals (mg/kg)				
Arsenic (As)	2.18	0.40	<0.10	<0.10
Mercury (Hg)	<0.05	<0.05	<0.05	<0.05
Lead (Pb)	2.54	<0.07	0.07	0.28

Values are expressed as mean ± SD where replicate measurements were available. Elemental concentrations are reported for the analyzed composite commercial plant materials. Instrumental detection limits were 0.10 mg/kg for As, 0.05 mg/kg for Hg, and 0.07 mg/kg for Pb. Values preceded by “<” indicate concentrations below the corresponding detection limit. General reference values of 10 mg/kg for As and Pb were considered for contextual comparison [20]. These analytical results do not represent independent commercial batches.

**Table 4:** Preliminary phytochemical screening of medicinal plants commercialized in Gamarra Market, Lima, Peru.

	Metabolites	*Aristeguietia ballii* (Asmachilca)	*Borago officinalis* (Borraja)	*Senecio canescens* (Oreja de Conejo)	*Eucalyptus globulus* (Eucalipto)
Ferric chloride	Phenolics compounds	(−)	(++)	(−)	(−)
Bornträger	Quinones	(−)	(−)	(−)	(+)
Baljet	Coumarins	(−)	(−)	(++)	(+++)
Kedde	Cardiac glycosides	(−)	(−)	(−)	(++)
Dragendorff	Alkaloids	(−)	(+)	(+)	(++)
Wagner	Alkaloids	(−)	(−)	(+)	(++)
Biuret	Proteins	(−)	(−)	(+++)	(−)
Sodium carbonate	Catechins	(−)	(−)	(+++)	(−)
Shinoda	Flavonoids	(−)	(+++)	(−)	(−)
Liebermann–Burchard	Steroids/Triterpenes	(−)	(−)	(−)	(+)
Mayer	Alkaloids	(−)	(−)	(−)	(+)
Fehling	Carbohydrates	(−)	(−)	(+++)	(+++)
Gelatine	Tannins	(+)	(+)	(++)	(+++)

(−), no detectable reaction; (+), weak positive reaction; (++), moderate positive reaction; (+++), strong positive reaction under the assay conditions. Reaction intensity represents a qualitative observation and should not be interpreted as quantitative evidence of metabolite concentration or compound-specific identification.

**Table 5:** Total phenolic content (TPC), total flavonoid content (TFC), and DPPH and ABTS radical-scavenging capacities of medicinal plant extracts prepared using solvents of different polarities.

Medicinal Plant	Extract	TPC (mg GAE/g DS)	TFC (mg QE/g DS)	DPPH Assay (mg TE/g DS)	ABTS Assay (mg TE/g DS)
*Aristeguietia ballii*	Chloroform	0.35 ± 0.00	7.46 ± 0.13	2.14 ± 0.04	3.04 ± 0.05
	Hydroethanolic	17.59 ± 0.31	0.06 ± 0.00	27.80 ± 0.50	31.51 ± 0.46
	Aqueous	37.45 ± 0.70	0.07 ± 0.00	18.99 ± 0.27	35.66 ± 0.64
*Borago officinalis*	Chloroform	0.12 ± 0.00	1.58 ± 0.03	1.57 ± 0.03	2.47 ± 0.05
	Hydroethanolic	25.75 ± 0.37	4.33 ± 0.08	33.39 ± 0.63	30.39 ± 0.55
	Aqueous	17.54 ± 0.31	7.52 ± 0.14	22.64 ± 0.41	41.01 ± 0.59
*Senecio canescens*	Chloroform	0.42 ± 0.00	1.68 ± 0.03	1.76 ± 0.03	4.80 ± 0.09
	Hydroethanolic	3.75 ± 0.07	8.80 ± 0.13	12.02 ± 0.22	48.59 ± 0.91
	Aqueous	0.42 ± 0.00	4.16 ± 0.07	12.79 ± 0.24	44.18 ± 0.80
*Eucalyptus globulus*	Chloroform	0.30 ± 0.00	21.92 ± 0.41	4.21 ± 0.08	8.17 ± 0.12
	Hydroethanolic	53.22 ± 1.00	6.16 ± 0.11	15.07 ± 0.22	57.42 ± 1.03
	Aqueous	7.07 ± 0.13	7.85 ± 0.12	22.82 ± 0.41	63.60 ± 1.19

Values are expressed as mean ± SD of three separately prepared extraction replicates (*n* = 3) obtained from independently weighed portions of the same homogenized composite commercial plant sample for each species–solvent combination. These replicates represent extraction-level experimental variability and do not constitute independent biological samples or commercial batches. TPC: total phenolic content, expressed as mg gallic acid equivalents (GAE)/g dry sample (DS); TFC: total flavonoid content, expressed as mg quercetin equivalents (QE)/g DS; DPPH and ABTS radical-scavenging capacities, expressed as mg Trolox equivalents (TE)/g DS.

## Data Availability

The data presented in this study are available within the article.

## Abbreviations

The following abbreviations are used in this manuscript:

ABTS	2,2′-Azino-bis(3-ethylbenzothiazoline-6-sulfonic acid)
DPPH	2,2-Diphenyl-1-picrylhydrazyl
TFC	Total Flavonoid Content
TPC	Total Phenolic Content
GAE	Gallic Acid Equivalents
QE	Quercetin Equivalents
TE	Trolox Equivalents
